# Supplementary material for: Validation of the Italian version of a patient-reported outcome measure for Hereditary Spastic Paraplegia
Source: PLoS One. 2024 Apr 1;19(4):e0301452. doi: 10.1371/journal.pone.0301452 (PMC10984402; doi:10.1371/journal.pone.0301452)
Supplement: S1 Checklist — (DOC) [file pone.0301452.s001.doc]

**COSMIN Reporting guideline for studies on measurement properties of patient reported outcome measures**

**Version August 2021**

**Joel J Gagnier^1-2^**

**Jianyu Lai^1^**

**Lidwine B. Mokkink^3^**

**Caroline B. Terwee^3^**

1. Department of Orthopaedic Surgery, University of Michigan, Ann Arbor, MI, USA

2. Department of Epidemiology, School of Public Health, University of Michigan, Ann Arbor, MI, USA

3. Amsterdam UMC, Vrije Universiteit Amsterdam, Department of Epidemiology and Data Science, Amsterdam Public Health research institute, Amsterdam, Netherlands

**Contact**

Dr. Joel J. Gagnier, jgagnier@umich.edu

Website: [www.cosmin.nl](http://www.cosmin.nl/)

| **General Reporting recommendations relevant for all studies on measurement properties** | | | | | |  |
| --- | --- | --- | --- | --- | --- | --- |
| **Item Number** | | **Item Name** | **Item Description** | | |  |
| **Report section: Title** | | |  | | |  |
| T1 | | Patient Reported Outcome Measure (PROM) | **Validation of the Italian Version of a Patient-Reported Outcome Measure for Hereditary Spastic Paraplegia** | | |  |
| T2 | | Measurement Property (MP) | Patient reported outcome collecting the patient’s personal evaluation of specific motor symptoms that can impact on walking ability. | | |  |
| T3 | | Study sample | We tested the Italian version of HSP-SNAP. The 12-item HSP-SNAP questionnaire was submitted to 20 external judges for comprehensibility and to 15 external judges for content validity assessment. We recruited 40 subjects with HSP and asked them to fill the questionnaire twice for test-retest procedure. We also recruited 44 healthy subjects who completed the HSP-SNAP once to test score variability. | | |  |
| **Report section: Abstract** | | |  | | |  |
| A1 | | PROM | “Hereditary Spastic Paraplegia-Self Notion and Perception Questionnaire” (HSP-SNAP). The HSP-SNAP is a patient-reported measure (PROM) focusing on 6 key features for HSP: stiffness, weakness, imbalance, reduced endurance, fatigue and pain. The aim of the HSP-SNAP is to assess the subjective impact of these symptoms on gait and walking performance. | | |  |
| A2 | | Measurement Property | HSP-SNAP questionnaire’s comprehensibility, content validity, reliability and correlation with other selected outcome measures (SPRS, 6MWT and SF-36). The correlation between HSP-SNAP and gender, patient’s age and age of disease onset. The comparison between symptoms “perceived” by HSP patients and those “perceived” by healthy subjects. | | |  |
| A3 | | Design | Study to assess comprehensibility and content validity and to assess test-retest reliability of the PROM | | |  |
| A4 | | Sample | For comprehensibility 20 judges. For content validity 15 judges. 40 patients with HSP were recruited for test-retest reliability. 44 healthy subjects. | | |  |
| A5 | | Methods | The 12-item HSP-SNAP questionnaire was submitted to 20 external judges for comprehensibility and to 15 judges for content validity assessment. We recruited 40 subjects with HSP and asked them to fill the questionnaire twice for test-retest procedure. They also completed the Medical Outcome Survey Short Form (SF-36) and were evaluated by the Spastic Paraplegia Rating Scale and the Six-Minute Walk Test. We also recruited 44 healthy subjects who completed the HSP-SNAP once to test score variability. | | |  |
| A6 | | Results | The HSP-SNAP content validity index was high (0.8±0.1) and the test-retest analysis showed high reliability (ICC= 0.94). The mean HSP-SNAP score (score range 0-48) of the HSP group was 22.2±7.8, which was significantly lower than healthy subjects (43.1±6.3). | | |  |
| A7 | | Discussion/Conclusions | The HSP-SNAP showed good validity and reliability and it could be used in combination with other objective outcome measures for clinical purposes or as endpoints for future clinical rehabilitation studies. | | |  |
| **Report section: Introduction** | | |  | | |  |
| I1 | | Name and describe the PROM of interest | We developed at the Scientific Institute Eugenio Medea in 2018 the “Hereditary Spastic Paraplegia-Self Notion and Perception Questionnaire” (hereinafter, HSP-SNAP), a disease-specific HSP-PROM, with the aim of collecting a patient’s personal evaluation of specific motor symptoms that can impact on walking ability. The HSP-SNAP is a patient-reported measure focusing on 6 key features for HSP: stiffness, weakness, imbalance, reduced endurance, fatigue and pain. The new tool consists of 12 items, 2 items for each dimension balanced in terms of positive/negative attitude to the question, to avoid “automatic compiling”. The total score is the sum of all item scores, with a maximum score of 48 (score range 0-48). The higher the score, the greater the individual well-being and the milder the symptoms. The HSP-SNAP was administered via paper and pencil. The time required for compilation was approximately 3-5 minutes, with a maximum of 10 minutes. | | |  |
| I2 | | Target population | All the symptoms explored by HSP-SNAP are regularly mentioned by patients, assessed by clinicians and reported in the literature. The aim of the HSP-SNAP is to assess the subjective impact of these symptoms on gait and walking performance. | | |  |
| I3 | | Citation for the original development of the PROM | We developed at the Scientific Institute Eugenio Medea in 2018 the “Hereditary Spastic Paraplegia-Self Notion and Perception Questionnaire” (hereinafter, HSP-SNAP), a disease-specific HSP-PROM, with the aim of collecting a patient’s personal evaluation of specific motor symptoms that can impact on walking ability. | | |  |
| I4 | | State of Knowledge & Rationale | Patient-reported outcome measures (PROMs) provide patients’ viewpoint about symptoms severity, functional and psychological problems, treatment satisfaction and quality of life, thus complementing objective outcome measures. PROMs have been used as outcomes in clinical trials for many rare diseases and could be useful for monitoring changes in disease natural history and disease progression. In his 8-year follow-up of 526 patients affected by spinocerebellar ataxias (SCAs) with different PROMs, Jacoby observed that patient-reported outcome measures could detect changes during disease progression. Generic PROMs are comparable across diseases but may miss important population-specific data as well as lack sensitivity and responsivity to disease specificities vs PROMs tailored to a specific rare condition. As Amprosi recently undelined, generic available PROMs are not suitable to detect change in HSP patients, and thus, he propose to create a tailored HSP-PROM that could be a significant integration to standard functional clinical outcomes. | | |  |
| I5 | | Definitions | No specialized terms are present | | |  |
| I6 | | Objectives and Hypotheses | Our first aim was to assess HSP-SNAP’s comprehensibility, content validity, reliability and correlation with other selected outcome measures. We asked a panel of external judges to assess the comprehensibility and the content validity of each item. Then, we evaluated the HSP-SNAP reliability with a test-retest procedure and looked at the correlation of HSP-SNAP with the SPRS, the 6MWT and the Medical Outcome Survey Short Form (SF-36).  Secondly, we decided to characterize the HSP sample recruited through the analysis of each single dimension explored by the questionnaire. We hypothesized that stiffness was the symptom most commonly perceived by patients, probably followed by weakness and imbalance and, last, pain and fatigue. We also explored a correlation between HSP-SNAP and gender, patient’s age and age of disease onset. Finally, we compared symptoms “perceived” by HSP patients and those “perceived” by healthy subjects (N=44 age-matched subjects) to collect data on the variability of scoring in a healthy population | | |  |
| **Report section: General Methods** | | |  | | |  |
| GM1 | | Study Design | The HSP-SNAP was evaluated by two panels of judges. For the comprehensibility assessment, we asked to 20 judges to verify if the items describe the competency explored in a complete and effective way, both from a structural and linguistic point of view. For the content validity assessment, we conducted short semi-structured interviews with 15 judges for their personal point of view on the HSP-SNAP symptoms’ selection. Afterwards, during a single-day evaluation at the hospital, patients were examined by experienced physical therapists by the Spastic Paraplegia Rating Scale (SPRS) and, if possible, the Six Minute Walking Test (6MWT).  The SPRS evaluates the multisystem involvement of HSP, covering in 13 items the wide spectrum of different aspects of the disease to evaluate its severity.  The 6MWT is used to evaluate walking endurance; it measures the walking distance covered in six minutes along a 25 m standardized path.  Additionally, patients completed the SF-36 and the HSP-SNAP.  The SF-36 is the most extensively validated and used outcome survey for quality of life. It provides a global view of health and is one of the most commonly used general PROM. It is made by 36 items that can be aggregated in 8 sub-scales and in 2 summary measures, i.e. physical component and mental component of health, according to Apolone et al. The HSP-SNAP was completed by patients with HSP a second time to test reliability. | | |  |
| GM2 | | Participants | The evaluation panel for comprehensibility included a group of clinical experts and two small representative samples of the HSP population and healthy subjects (20 subjects). For content validity, we selected a panel of clinical experts and patients with HSP (15 subjects). 40 patients with HSP were recruited following these diagnostic clinical criteria: 1) “pure” or “complicated” genetically determined HSP or 2) not genetically determined paraplegia with the exclusion of all other causes of spasticity through brain and spinal cord imaging studies, metabolic analysis, clinical history data (i.e. pregnancy and or/ delivery complication, Apgar evaluation at birth).  Inclusion criteria were: age above 9 years, ability to walk at least 10 meters (a walking device was allowed). Exclusion criteria were: wheelchair-bound patients, patients with severe orthopedic conditions, cardiovascular dysfunction, psychopathological symptoms, moderate/severe mental retardation or cognitive decline. Healthy subjects were recruited according to the following inclusion criteria: age above 9 years and being in good health (44 subjects). | | |  |
| GM3 | | PROM administration | The HSP-SNAP was evaluated by two panels of judges. For comprehensibility, we asked judges to verify if the items describe the competency explored in a complete and effective way, both from a structural and linguistic point of view. Each item could be judged as totally comprehensible, partially comprehensible or not comprehensible.  For content validity, we conducted short semi-structured interviews with judges for their personal point of view on the HSP-SNAP symptoms’ selection. Each item could be valued as essential/necessary, useful but not essential, or not essential/necessary.  The HSP-SNAP was administered via paper and pencil. No time limit for the compilation. Participants received a clear explanation by the evaluator but could ask for clarifications, if needed, and we record difficulties, if any, in understanding and completing each single item. | | |  |
| GM4 | | Data collection procedures | The HSP-SNAP was completed on two consecutive days to assess its reliability. Participants were not aware that this was a test-retest evaluation and we explicitly stated that the second test was designed to evaluate their current status. Afterwards, during a single-day evaluation at the hospital, patients were examined by experienced physical therapists by SPRS and, if possible, 6MWT. Additionally, patients completed the SF-36 (the Italian version of Apolone et al. 1997) and the HSP-SNAP.  Healthy subjects filled the HSP-SNAP form once with the purpose to define normative data. | | |  |
| GM5 | | Power/sample size calculation | To estimate the sample size, an agreement between two measures of HSP-SNAP on two consecutive days (see Study Protocol) was selected as primary outcome measure. Given an acceptable reliability level of 0.6, an expected reliability of 0.8, a power of 0.80 with alpha=0.05, it was estimated that the number needed to verify the agreement was 39 patients (33, 34). G*Power 3.1.9.4 was used to calculate the sample size. | | |  |
| GM6 | | Statistical analyses | To assess item comprehensibility, for each item we determined to what percentage of judges declared the item totally comprehensible. To assess the *content validity ratio (CVR),* the data collected by judges were analyzed item-by-item according to Lawshe (1975).  We then computed the *content validity index* (CVI) for the whole test, simply calculating the mean CVR values of items.  The normality of the distribution was assessed for all the variables by means of Shapiro-Wilk test and the following analyses were defined accordingly.  The HSP-SNAP reliability was assessed for possible systematic errors by means of a paired T-test and calculating the intraclass correlation coefficient (ICC) for absolute agreement between the two measures collected on two consecutive days in the 40 subjects with HSP. Furthermore, the Standard Error of Measurement (SEM) and the Minimum Detectable Change (MDC) were computed, according to Polit. The Pearson correlation was computed between the two measures of HSP-SNAP.  Furthermore, potential relations between the HSP-SNAP and SPRS, 6MWT and SF-36 were explored by Pearson correlation. The HSP group was compared with the healthy group in terms of age (by means of a Mann-Whitney U test), and gender (Chi-squared test). The mean HSP-SNAP scores were computed in the two groups and compared by means of a Mann-Whitney U test. Furthermore, the correlation between the HSP-SNAP scores and age was assessed by means of Spearman rank correlation in the two groups. The possible influence of gender was assessed with a Chi-squared test. In the HSP group, the correlation of HSP-SNAP with age at disease onset was further evaluated. To look for potential differences between pure and complicated HSP, a Mann-Whitney U test was applied to compare HSP-SNAP scores in patients with pure and complicated HSP.  Finally, we analyzed each of the six dimensions (i.e. stiffness, weakness, imbalance, reduced endurance, fatigue and pain) explored by the questionnaire. We computed the mean between the two items related to the same dimension and compared them by means of a repeated measure ANOVA and Bonferroni-corrected T test as post-hoc analysis. To conclude, we computed the Pearson correlation between the total HSP-SNAP score and the six dimensions. | | |  |
| GM7 | | Missing data | All the 40 patients with HSP completed the HSP-SNAP questionnaire twice. No missing items were detected. | | |  |
| GM8 | | Post hoc analysis | No post hoc analysis are reported. | | |  |
| **Report section: General Results** | | |  | | |  |
| GR1 | | Missing data | All the 40 patients with HSP completed the HSP-SNAP questionnaire twice. No missing items were detected.  Only 35 patients were able to perform the 6MWT. Furthermore, we had 5 missing data for SF-36 due to both young age and/or data loss. | | |  |
| GR2 | | Participant/patient Characteristics | 20 judges were recruited to assess the comprehensibility of HSP-SNAP. The judges were 10 physical therapists with work experience on average of 15 years, 5 patients with HSP and 5 healthy subjects. 15 judges were recruited to assess content validity of HSP-SNAP. The judges were 10 expert clinicians (resident neurologist, resident physiatrist and physical therapist; work experience on average 14 years) and 5 patients affected by HSP. According to Lawshe, the required minimum value of CVR was 0.49, considering 15 judges. 40 patients with HSP were recruited according to the sample size estimation. The molecular diagnosis was available in 24 of 40 patients (10 were carrying mutation in the SPG4; 4 in SPG7; 2 in SPG3A; 2 in SPG11; 1 in SPG5; 1 in SPG8; 1 in SPG30; 1 in SPG31; 1 in SPG35; 1 in SPG72). 27 patients were affected by a pure HSP, whereas 13 patients were affected by a complicated form (with an addition of other neurological and non-neurological symptoms, such as cerebellar ataxia, peripheral neuropathy, retinopathy and mild cognitive impairment). 4 out of 40 patients were showing a mild mental retardation but had the ability to critically answer to the questions. 4 out of 40 patients were regularly taking antispastic medication. 3 out of 40 were taking antidepressant drugs.  44 healthy subjects were enrolled in the study to have a sample size comparable to that of patients with HSP. The two groups (healthy subjects vs subjects with HSP) were comparable in terms of age (p=.468) but not in terms of gender (p=.025), being the group of patients with a prevalence of males. | | |  |
| GR3 | | Sample size | 20 judges were recruited to assess the comprehensibility of HSP-SNAP. 15 judges were recruited to assess content validity of HSP-SNAP. 40 patients with HSP were recruited according to the sample size estimation. 44 healthy subjects were enrolled in the study to have a sample size comparable to that of patients with HSP. | | |  |
| **Report section: Discussion** | | |  | | |  |
| D1 | | MP evidence | Results showed optimal comprehensibility and globally good scores of content validity ratio (CVR) for each single item and a high CVI score, confirming a good total content validity. The judges judged the questionnaire as complete and did not suggest including any other items.  The HSP-SNAP test-retest reliability and the high test-retest reliability value (ICC) confirmed that the HSP-SNAP is a robust measure of subjective symptom perception in the HSP population. We also tested the HSP-SNAP correlation with SPRS, 6MWT and SF-36 and, as expected, we did not find any correlation either with SPRS or with 6MWT. Instead, a moderate correlation between HSP-SNAP and both the physical component and the mental component of SF-36 emerged.  The lack of correlations with both SPRS and 6MWT is not surprising, given the way the questionnaire was designed and the different dimensions tapped. The lack of correlation is not a limitation. It provides further evidence that a patient’s point of view may be different from what is usually seen in a clinical setting and must be considered along clinical objective measures. The moderate correlation between HSP-SNAP and SF-36, which collects subjective evaluations of both motor functional impairments and psychological well-being, supports the fact that the HSP-SNAP measures the subjective impact of HSP-related symptoms. Our questionnaire highlights how objective and subjective evaluations can differ in chronic neurologic disorders and underlines the relevance of patient perception.  Our results support our assumption, showing that the primary aspects of this disease perceived by patients is stiffness, resulting in lack of gait smoothness, followed by lack of strength and impaired balance. On the other hand, reduced endurance, fatigue and pain seem to be perceived as less disabling in walking.  Finally, we compared “perceived” symptoms in our HSP group and in healthy subjects. Data showed a statistically significant difference between scores in these two populations, with healthy subjects scoring twice as much HSP subjects. This proves the sensitivity of the HSP-SNAP in measuring changes due to pathology. Only healthy subjects reached the maximum score of 48 points, whereas in the HSP population, HSP-SNAP seems to be relevant in all diseases stages with an almost normal distribution without score of 0 and 48 points preventing a floor and ceiling effect. | | | |
| D2 | | Practical relevance | The HSP-SNAP is the first assessment measure, validated in its Italian version comprehensive of all the most significant motor signs and symptoms that could affect walking ability in the HSP population. It can be completed in less than 10 minutes and does not need any special setting or equipment. Moreover, it focuses on the patient as stakeholder in the evaluation process. | | | |
| D3 | | Strengths and limitations | The HSP-SNAP questionnaire can be regularly used in combination with other objective outcome measures either for clinical purposes or as endpoints for future clinical rehabilitation studies, to ensure that individuals with HSP receive proper help for their needs. This work has some limitations. First of all, patients’ heterogeneity: a broad spectrum of different genotypes and phenotypes of HSP patients was included in our analysis. One third of our HSP population had a complicated HSP with signs and symptoms different from those tapped in the HSP-SNAP, such as visual impairment or peripheral neuropathy that could further affect patients’ walking ability. Nevertheless, being HSP a relatively rare disease, we believe that 40 patients make a good sample size.  Additionally, we did not collect specific information on mood or anxiety disorders potentially influencing the assessment and we assumed that the presence of depressive symptoms or symptoms of anxiety could be correlated with systematically lower scores of the instrument. Despite these limitations, this study has several strengths. | | | |
| D4 | | Generalizability | HSP-SNAP questionnaire can be used for both “pure” and “complicated” forms of HSP patients. It cannot be generalized to other diseases being a disease-specific PROM. | | | |
| D5 | | Instrument changes | No changes needed. | | | |
| D6 | | Future Research | Future studies should test the English version of the HSP-SNAP in an English-speaking HSP population and may help validate the HSP-SNAP in clinical studies with independent and larger patient cohorts in view of a wider clinical use. | | | |
| **Report section: Conclusions** | | |  | | | |
| C1 | | Conclusions | The HSP-SNAP questionnaire can be regularly used in combination with other objective outcome measures either for clinical purposes or as endpoints for future clinical rehabilitation studies, to ensure that individuals with HSP receive proper help for their needs. The HSP-SNAP may also support communication between patients and clinicians as well as shared decision-making in the rehabilitation process. | | | |
| **Report section: Other information** | | |  | | | |
| O1 | | Conflict of Interest | No conflict of interest are declared. | | | |
| **Specific Reporting recommendations for studies on Content Validity** | | | | |  | |
| **Item Number** | **Item Name** | | | **Item Description** |  | |
| CV1 | Relevance | | | For content validity, we conducted short semi-structured interviews with judges for their personal point of view on the HSP-SNAP symptoms’ selection. Each item was valued as essential/necessary, useful but not essential, or not essential/necessary. |  | |
| CV2 | Comprehensiveness | | | With a short semi-structured interview, we asked the panel whether all key concepts were included. |  | |
| CV3 | Comprehensibility | | | With a short semi-structured interview, we asked the panel if the HSP-SNAP instructions, the items and the response options were comprehensible. |  | |
| CV4 | Relevance results | | | All items were considered relevant for the construct, population, and context of use of interest by patients and/or professionals. |  | |
| CV5 | Response options and recall period | | | Response options and recall period were considered appropriate by patients and professionals |  | |
| CV6 | Comprehensiveness results | | | All the key concepts were included in the HSP-SNAP. |  | |
| CV7 | Comprehensibility results | | | Patients understood the HSP-SNAP instructions, items, and response options as intended. |  | |

| **Specific Reporting recommendations for studies on Reliability** | | |
| --- | --- | --- |
| **Item Number** | **Item Name** | **Item Description** |
| R1 | PROM Administrations | The HSP-SNAP was self-administered via paper and pencil. It is a short questionnaire that can be completed in less than 10 minutes and does not need any special setting or equipment. Participants received a clear explanation by the evaluator but could ask for clarifications, if needed, and difficulties, if any, in understanding and completing each single item were recorded.  The HSP-SNAP was completed again on the following day to assess its reliability. Participants were not aware that this was a test-retest evaluation and we explicitly stated that the second test was designed to evaluate their current status. Healthy subjects filled the HSP-SNAP form only once. |
| R2 | Statistical analyses | The HSP-SNAP reliability was assessed for possible systematic errors by means of a paired T-test and calculating the intraclass correlation coefficient (ICC) for absolute agreement between the two measures collected on two consecutive days in the 40 subjects with HSP. Furthermore, the Standard Error of Measurement (SEM) and the Minimum Detectable Change (MDC) were computed, according to Polit [37]. The Pearson correlation was computed between the two measures of HSP-SNAP. In the test-retest reliability no systematic errors between the two measures emerged on the T-test (p=.476; t -.720). Furthermore, with an ICC=0.94, the HSP-SNAP showed an optimal test-retest reliability. The SEM was 1.7, which means that approximately two points in the HSP-SNAP were due to the scale unreliability. The MDC was 4.8. The two measures of HSP-SNAP showed a good correlation (r=.883, p<.001). |
| R3 | Methods to improve reliability | The reliability was very high and no measures to improve it are needed |
