# Supplementary material for: Validation of the Italian version of a patient-reported outcome measure for Hereditary Spastic Paraplegia
Source: PLoS One. 2024 Apr 1;19(4):e0301452. doi: 10.1371/journal.pone.0301452 (PMC10984402; doi:10.1371/journal.pone.0301452)
Supplement: S1 Table — (DOCX) [file pone.0301452.s002.docx]

**Questionario Self-Notion and Perception nella Paraparesi Spastica Ereditaria**

**(HSP- SNAP)**

Fortemente in disaccordo

In disaccordo

Neutrale

D'accordo

Totalmente d'accordo

1

2

3

4

5

1

2

3

4

5

12 – Ho avuto buon equilibrio nel cammino.

11 – La fatica fisica ha limitato le mie attività motorie quotidiane.

Fortemente in disaccordo

In disaccordo

Neutrale

D'accordo

Totalmente d'accordo

Fortemente in disaccordo

In disaccordo

Neutrale

D'accordo

Totalmente d'accordo

1

2

3

4

5

1

2

3

4

5

10 – Ho eseguito le mie attività motorie quotidiane senza dolore fisico.

9 – Ho avuto scarso equilibrio nel cammino.

Fortemente in disaccordo

In disaccordo

Neutrale

D'accordo

Totalmente d'accordo

Fortemente in disaccordo

In disaccordo

Neutrale

D'accordo

Totalmente d'accordo

1

2

3

4

5

1

2

3

4

5

8 – Ho avuto le gambe forti nel cammino.

7 – La scarsa resistenza nel cammino mi ha ostacolato.

Fortemente in disaccordo

In disaccordo

Neutrale

D'accordo

Totalmente d'accordo

Fortemente in disaccordo

In disaccordo

Neutrale

D'accordo

Totalmente d'accordo

1

2

3

4

5

1

2

3

4

5

6 – Mi sono sentito fluido/sciolto nel cammino.

5 – Il dolore fisico mi ha ostacolato nelle mie attività motorie quotidiane.

Fortemente in disaccordo

In disaccordo

Neutrale

D'accordo

Totalmente d'accordo

Fortemente in disaccordo

In disaccordo

Neutrale

D'accordo

Totalmente d'accordo

1

2

3

4

5

1

2

3

4

5

4 – Ho avuto una buona resistenza nel cammino.

3 –La debolezza delle gambe ha limitato il mio cammino.

Fortemente in disaccordo

In disaccordo

Neutrale

D'accordo

Totalmente d'accordo

Fortemente in disaccordo

In disaccordo

Neutrale

D'accordo

Totalmente d'accordo

1

2

3

4

5

1

2

3

4

5

2 – Ho svolto le mie attività motorie quotidiane senza fatica fisica.

1 - La rigidità delle gambe ha influenzato il mio cammino.

Fortemente in disaccordo

In disaccordo

Neutrale

D'accordo

Totalmente d'accordo

Scelga un punteggio per ogni affermazione.

Nell’ultima settimana:
