# Supplementary material for: Validation of the Italian version of a patient-reported outcome measure for Hereditary Spastic Paraplegia
Source: PLoS One. 2024 Apr 1;19(4):e0301452. doi: 10.1371/journal.pone.0301452 (PMC10984402; doi:10.1371/journal.pone.0301452)
Supplement: S1 File — (DOCX) [file pone.0301452.s003.docx]

**DETTAGLIO PROGETTO DI RICERCA**

| **TITOLO del PROGETTO:** | SNAP: Misura della percezione soggettiva del sintomo nelle paraparesi spastiche ereditarie (HSP) |
| --- | --- |
| **RESPONSABILE SCIENTIFICO:** | DIELLA, Eleonora |
| **POLO:** | Bosisio Parini (LC) |
| **CAPOFILA=PROMOTORE:** | IRCCS E. MEDEA – Sez. Scientifica dell’Associazione “La Nostra Famiglia” |
| **CENTRI COINVOLTI:** | IRCCS E. MEDEA Polo di Conegliano (TV) e Brindisi (BR) |
| **BANDO DI RIFERIMENTO:** | Ricerca Spontanea |
| **AREA di RICERCA:** | Tecnologie applicate (neuroimaging, bioingegneria, robotica), organizzazione e gestione dei servizi sanitari |
| **DURATA:** | 24 MESI |

**DESCRIZIONE DEL PROGETTO**

Le paraparesi spastiche ereditarie (prevalenza 1.8-9.8/100.000) sono un gruppo geneticamente e clinicamente eterogeneo di disordini neurodegenerativi causati da degenerazione retrograda delle fibre assonali cortico-spinali (forme “pure”)(1). Il coinvolgimento anche di altre componenti del sistema nervoso centrale o di altri sistemi viene descritto nelle “forme complicate”(2).

Solitamente questa patologia inizia a manifestarsi con alterazioni della marcia dovute a incremento del tono spastico a livello degli arti inferiori, successivamente il declino funzionale può essere legato in misura variabile alla riduzione della forza muscolare e al peggioramento dell’equilibrio con conseguente riduzione dell’endurance e incremento dell’affaticabilità (3). Nelle forme avanzate le autonomie di spostamento si riducono drasticamente fino alla necessità della carrozzina come modalità preferenziale di spostamento.

Ad oggi, non esiste una cura farmacologica per questa patologia.

Le evidenze in letteratura rispetto all’efficacia di strumenti riabilitativi volti alla cura del sintomo nel soggetto con paraparesi spastica ereditaria sono scarse con trial clinici caratterizzati da *sample size* ridotti e misure di *outcome* non comparabili(4-6). Diventa quindi di estrema importanza per valutare l’efficacia di potenziali nuovi trattamenti l’utilizzo di misure di outcome appropriate in termini di requisiti psicometrici (7).

Il questionario Self-Notion and Perception (SNAP) sviluppato presso l’IRCCS “E. Medea” da Eleonora Diella e Roberta Morganti, nasce dall’esigenza di quantificare la percezione soggettiva del paziente affetto da HSP dei sintomi tipici di patologia, quali spasticità, debolezza, alterazioni dell’equilibrio, resistenza nel cammino, dolore e fatica.

Scopo di questo studio è validare questo strumento e testarne l’affidabilità, ricercando correlazioni con le scale di misura utilizzate in letteratura per la valutazione del paziente con HSP.

Le più utilizzate per questa popolazione sono la Spastic Paraplegia Rating Scale (SPRS) e il Six-Minute Walk Test (6MWT) che valutano rispettivamente gravità della patologia e livello di endurance (8-9).

Il questionario SNAP oltre a darci informazioni su un aspetto clinico ancora non indagato, cioè il giudizio critico del paziente rispetto alla sua sintomatologia, potrebbe essere utilizzato in abbinamento ad altre misure di *outcome* come *endpoint* in futuri trial clinici di carattere riabilitativo.

Nello strutturare questo progetto siamo ben coscienti che la correlazione tra il dato soggettivo ed il dato oggettivo non è così scontata e che la valutazione soggettiva del paziente potrà essere “sporcata” dalla componente emotiva.

**Tipologia di progetto**

Tecnologie abilitanti

Studio spontaneo - studio prospettico

**Aspetti metodologici**

**Background**: La necessità di sperimentare nuove proposte terapeutiche richiede l’utilizzo di misure di *outcome* valide, affidabili, sensibili e semplici da somministrare. In letteratura non esiste alcuna misura di *outcome* che mira a valutare la percezione critica del paziente con HSP rispetto alla sua sintomatologia; a fronte in realtà di varie scale che rilevano il dato oggettivo come la Modified Ashworth Scale(MAS) per misurare il tono muscolare, la scala di valutazione della forza muscolare del Medical Research Council (MRC), la scala SPRS per definire la gravità della patologia ed il 6MWT per valutare l’endurance (1-3).

BIBLIOGRAFIA

1. Bohannon, R.W. (1987). Interrater reliability of a modified Ashworth scale of muscle spasticity. Phys Ther, 67,206-7
2. Medical Research Council. Aids to the examination of the peripheral nervous system, Memorandum no. 45, Her Majesty's Stationery Office, London, 1981.
3. Martinuzzi, A. (2016) Clinical and Paraclinical Indicators of Motor System Impairment in Hereditary Spastic Paraplegia: A Pilot Study. PLOS ONE, 11 (4)

**Obiettivo principale:** Testare la validità, indagando la correlazione con Spastic Paraplegia Rating Scale e con Six- Minute Walk Test, e l’affidabilità (mediante modalità test-retest) del questionario SNAP nella popolazione suddetta.

**Obiettivo secondario:** Analizzare la dimensione della percezione soggettiva del paziente affetto da HSP dei sintomi tipici di patologia, facendo un’analisi media (o stratificata per patologia) dei *sotto-item* della scala. Confrontare i risultati ottenuti con SNAP su popolazione affetta da HSP rispetto ai sani.

**Note sul calcolo della dimensione campionaria:**Ci si aspetta che la correlazione fra SNAP e SPRS – se presente - sia moderata (fra 0.45 e 0.6), poiché la scala SPRS misura anche componenti non misurate da SNAP, cioè parametri oggettivi di valutazione sia selettiva e che funzionale. Considerando dunque il caso più negativo (correlazione 0.45), alfa=0.05, potenza=0.90, si stima che siano necessari 39 pazienti per verificare la presenza di una correlazione.

Con questa dimensione del campione, inoltre, sarà anche possibile evidenziare differenze fra sani e pazienti con una potenza del 99%. Infatti, ipotizzando per i pazienti una media del punteggio SNAP di 30 (con deviazione standard di 12) e per i sani una media del punteggio SNAP di 40 (con deviazione standard di 7), sono necessari 32 soggetti per ciascun gruppo al fine di evidenziare differenze con alfa=0.05 e beta=0.01.

**Attività previste:**

- 1^Fase, RECLUTAMENTO: saranno reclutati nel Polo di Bosisio Parini, di Conegliano Veneto e di Brindisi **40 soggetti** affetti da paraparesi spastica ereditaria geneticamente determinata o soggetti senza genetica definita ma che mostrano inequivocabilmente al momento della valutazione una famigliarità dominante o recessiva con coinvolgimento esclusivo del sistema piramidale (forma diagnosticata per famigliarità accertata ed esclusione di altre patologie similari).

Verranno poi reclutati anche **40 soggetti sani** a cui verrà sottoposto il questionario per valutare la variabilità del punteggio all’interno di una popolazione sana;

- 2^ Fase, VALUTAZIONE: il questionario SNAP sarà somministrato 2 volte in 2 giorni consecutivi per valutarne l’affidabilità. Il questionario verrà auto compilato dal paziente. Poi sarà somministrata la scala SPRS e, laddove possibile, il 6MWT.

**Risultati attesi:** la validazione di questo questionario e la verifica della sua affidabilità ci permetteranno di utilizzare questo strumento come *outcome measure* in futuri trial clinici con lo scopo di valutare i benefici ottenuti da nuovi trattamenti riabilitativi. La soggettività di questo strumento potrebbe non correlare adeguatamente con l’oggettività degli altri strumenti di misura (SPRS e 6MWT) somministrati.

#### Descrizione della popolazione

**Caratteristiche dei soggetti/pazienti:**

- 40 pazienti affetti da paraparesi spastica ereditaria (HSP) geneticamente determinata o soggetti senza genetica definita ma che mostrano inequivocabilmente al momento della valutazione una famigliarità dominante o recessiva con coinvolgimento esclusivo del sistema piramidale (forma diagnosticata per famigliarità accertata ed esclusione di altre patologie similari);
- 40 soggetti sani in buone condizioni di salute in relazione all'età.

**N. totale soggetti/pazienti:** 80

**N. soggetti/pazienti per centro:** La maggior parte dei soggetti affetti sarà reclutata nel Polo di Bosisio Parini, in misura minore presso i Poli di Conegliano Veneto e Brindisi. Tutti i soggetti sani saranno reclutati presso il polo di Bosisio Parini.

**Giustificazione della dimensione del campione:** i calcoli statistici della dimensione campionaria individuano in 39 il numero minimo di pazienti con HSP per verificare la presenza di correlazione con le altre misure di *outcome*.

**Criteri di inclusione:** età >9 anni, QI >80 (avere un cognitivo non integro a causa di decadimento o ritardo mentale può rappresentare un limite). Il paziente deve essere in grado di camminare per almeno 10 mt. In interni, anche con ausilio. Soggetti sani "*age-matched*" alla popolazione con HSP.

**Criteri di esclusione:** età <9 anni, QI <80, perdita della deambulazione, aspetti psicopatologici che possono inficiare la validità del dato raccolto.

**Criteri generali di valutazione dell’efficacia:**NP

**Criteri generali di valutazione della tollerabilità:** la somministrazione del questionario SNAP richiede circa 10 minuti, quindi si reputa sia assolutamente tollerabile per il paziente.

**Metodologia statistica: indicare % di drop-out attesi**: la SPRS e il 6MWT sono normalmente somministrati a pazienti con HSP. Si ipotizza che la richiesta di effettuare la valutazione aggiuntiva con il questionario SNAP non causi non aderenza allo studio.

#### Valutazione del rapporto rischio/beneficio

**Possibili vantaggi:** Definire uno strumento che sia valido ed affidabile da utilizzare in futuri trial clinici di carattere riabilitativo.

**Possibili svantaggi e rischi:** Nulli

**Alternative diagnostico-terapeutiche**: NP

**Procedure dello studio:** NP

**Misure precauzionali a salvaguardia dei soggetti/pazienti:** Assenti

**Valutazione complessiva del rapporto rischio beneficio:** Positivo. Nullo il rischio a fronte del beneficio di avere uno strumento utile da utilizzare in futuri trial clinici riabilitativi.

BIBLIOGRAFIA

(1)Fink, J.K.(2003). The hereditary spastic paraplegias. Arch Neurol,60,1045-1049.

(2)Appleton, R.(1991). “Pure” and “complicated “forms of hereditary spastic paraplegia presenting in childhood. Dev Med Child Neurol,33,304-312

(3)Marsden, J.(2012). Muscle paresis and passive stiffness: Key determinants in limiting function in Hereditary and Sporadic Spastic Paraparesis. Gait &Posture,35,266-271

(4)de Niet, M.(2015).Functional effects of botulinum toxin type-A treatment and subsequent stretching of spastic calf muscles: a study in patients with hereditary spastic paraplegia. J Rehabil Med,47,147-153

(5)Bertolucci, F.(2015). Robotic gait training improves skills and quality of life in hereditary spastic paraplegia. NeuroRehabilitation,36,93-99

(6)Zhang, Y.(2014) The effect of hydrotherapy treatment on gait characteristics of hereditary spastic paraparesis patients. Gait & Posture,39,1074-1079

(7)Michels, E(1982). Evaluation and research in physical therapy. Phys Ther,62,828-834

(8)Schule, R(2006). The Spastic Paraplegia Rating Scale (SPRS). Neurology,67,430-434

(9)ATS(2002). Statement: Guidelines for the Six-Minute Walk Test (6MWT). Am J Respir Crit Care Med,166,111-11
