# Supplementary material for: Validation of the Italian version of a patient-reported outcome measure for Hereditary Spastic Paraplegia
Source: PLoS One. 2024 Apr 1;19(4):e0301452. doi: 10.1371/journal.pone.0301452 (PMC10984402; doi:10.1371/journal.pone.0301452)
Supplement: S2 File — (DOCX) [file pone.0301452.s004.docx]

**RESEARCH PROJECT**

| **TITLE:** | SNAP: Measurement of subjective symptom perception in hereditary spastic paraparesis (HSP) |
| --- | --- |
| **PRINCIPAL INVESTIGATOR:** | Dr. DIELLA Eleonora |
| **HEADQUARTER:** | Bosisio Parini (LC) |
| **PROMOTER:** | IRCCS E. MEDEA – Scientific Institute |
| **PARTNERS:** | IRCCS E. MEDEA Headquarter of Conegliano (TV) e Brindisi (BR) |
| **REFERENCE NOTICE:** | Spontaneous Search |
| **RESEARCH AREA:** | Applied technologies (neuroimaging, bioengineering, robotics), organization and management of health services |
| **DURATION:** | 24 months |

**PROJECT DESCRIPTION.**

Hereditary spastic paraparesis (prevalence 1.8-9.8/100,000) is a genetically and clinically heterogeneous group of neurodegenerative disorders caused by retrograde degeneration of cortico-spinal axonal fibers ("pure" forms)(1). Involvement also of other components of the central nervous system or other systems is described in the "complicated forms"(2).

Usually this pathology begins to manifest with alterations in walking due to increased spastic tone in the lower limbs, thereafter functional decline may be related to varying degrees to reduced muscle strength and worsened balance resulting in reduced endurance and increased fatigability (3). In advanced forms, movement autonomies are drastically reduced to the point where wheelchairs are needed as the preferred mode of movement.

To date, there is no pharmacological treatment for this condition.

Evidence in the literature with respect to the efficacy of rehabilitation tools aimed at symptom treatment in the subject with hereditary spastic paraparesis is scarce with clinical trials characterized by small sample sizes and non-comparable outcome measures(4-6). Therefore, it becomes of utmost importance, to evaluate the effectiveness of potential new treatments, to use appropriate outcome measures in terms of psychometric requirements (7).

The Self-Notion and Perception (SNAP) questionnaire developed at the IRCCS "E. Medea" by Eleonora Diella and Roberta Morganti, was born from the need to quantify the subjective perception of the patient with HSP of typical symptoms of pathology, such as spasticity, weakness, balance alterations, walking resistance, pain and fatigue.

The purpose of this study is to validate this instrument and test its reliability by searching for correlations with measurement scales used in the literature to assess the patient with HSP.

The most widely used for this population are the Spastic Paraplegia Rating Scale (SPRS) and the Six-Minute Walk Test (6MWT), which assess severity of pathology and level of endurance, respectively (8-9).

The SNAP questionnaire in addition to giving us information on a yet uninvestigated clinical aspect, i.e., the patient's critical judgment of his or her symptomatology, could be used in conjunction with other outcome measures as an endpoint in future clinical trials of a rehabilitative nature.

In structuring this project we are well aware that the correlation between subjective and objective data is not so obvious and that the patient's subjective assessment may be "soiled" by the emotional component.

**Type of project**

Enabling technologies

Spontaneous study-prospective study

**Methodological Aspects.**

Background: The need to test new therapeutic proposals requires the use of outcome measures that are valid, reliable, sensitive, and easy to administer. In the literature, there is no outcome measure that aims to assess the critical perception of the patient with HSP with respect to his or her symptomatology; compared in fact with various scales that detect the objective data such as the Modified Ashworth Scale (MAS) to measure muscle tone, the Medical Research Council (MRC) muscle strength rating scale, the SPRS scale to define the severity of the pathology and the 6MWT to assess endurance (1-3).

REFERENCE

1. Bohannon, R.W. (1987). Interrater reliability of a modified Ashworth scale of muscle spasticity. Phys Ther, 67,206-7
2. Medical Research Council. Aids to the examination of the peripheral nervous system, Memorandum no. 45, Her Majesty's Stationery Office, London, 1981.
3. Martinuzzi, A. (2016) Clinical and Paraclinical Indicators of Motor System Impairment in Hereditary Spastic Paraplegia: A Pilot Study. PLOS ONE, 11 (4)

Primary outcome: To test the validity, by investigating the correlation with Spastic Paraplegia Rating Scale and Six- Minute Walk Test, and reliability (by test-retest mode) of the SNAP questionnaire in the above population.

Secondary outcome: To analyze the dimension of the HSP patient's subjective perception of typical symptoms of pathology by doing an average analysis (or stratified by pathology) of the sub-items of the scale. To compare the results obtained with SNAP on population with HSP versus healthy people.

Notes on sample size calculation**:** The correlation between SNAP and SPRS-if present-is expected to be moderate (between 0.45 and 0.6), since the SPRS scale also measures components not measured by SNAP, i.e., objective parameters of both selective and functional assessment. Thus, considering the most negative case (correlation 0.45), alpha=0.05, power=0.90, an estimated 39 patients are needed to test for correlation.

With this sample size, it will also be possible to show differences between healthy and patients with a power of 99%. In fact, assuming for patients a mean SNAP score of 30 (with standard deviation of 12) and for healthy people a mean SNAP score of 40 (with standard deviation of 7), 32 subjects from each group are needed in order to show differences with alpha=0.05 and beta=0.01.

**Planned activities:**

**-** 1^Phase, RECRUITMENT: 40 subjects with genetically determined hereditary spastic paraparesis or subjects without defined genetics but who unequivocally show at the time of evaluation a dominant or recessive familiarity with exclusive involvement of the pyramidal system (form diagnosed by established familiarity and exclusion of other similar pathologies) will be recruited at the Bosisio Parini, Conegliano Veneto and Brindisi Polo. Forty healthy subjects will then also be recruited and given the questionnaire to assess the variability of the score within a healthy population;

**-** 2nd Phase, EVALUATION: The SNAP questionnaire will be administered 2 times on 2 consecutive days to assess its reliability. The questionnaire will be self-completed by the patient. Then the SPRS scale and, where possible, the 6MWT will be administered.

#### Expected results: validation of this questionnaire and verification of its reliability will allow us to use this instrument as an outcome measure in future clinical trials with the aim of assessing the benefits obtained from new rehabilitation treatments. The subjectivity of this instrument may not correlate adequately with the objectivity of the other measurement instruments (SPRS and 6MWT) administered.

**Description of the population**

Characteristics of subjects/patients:

- 40 patients with genetically determined hereditary spastic paraparesis (HSP) or subjects without defined genetics but who unequivocally show at the time of evaluation a dominant or recessive familial pattern with exclusive involvement of the pyramidal system(form diagnosed by established familial pattern and exclusion of other similar diseases);

- 40 age-matched healthy subjects.

Total no. of subjects/patients: 80

No. subjects/patients per center: Most affected subjects will be recruited at the Bosisio Parini Pole, to a lesser extent at the Conegliano Veneto and Brindisi Poles. All healthy subjects will be recruited at the Bosisio Parini hub.

Sample size justification: statistical sample size calculations identify 39 as the minimum number of patients with HSP to test for correlation with other outcome measures.

Inclusion criteria: age >9 years, IQ >80 (having non-intact cognitive due to mental impairment or retardation may be a limitation). Patient must be able to walk at least 10 mt. Indoor, even with aid. Healthy subjects "aged-matched" to the population with HSP.

Exclusion criteria: age <9 years, IQ <80, loss of ambulation, psychopathological aspects that may affect the validity of the collected data.

#### General evaluation criteria for efficacy: NP

#### General criteria for assessing tolerability: administration of the SNAP questionnaire takes about 10 minutes, so it is believed to be completely tolerable for the patient.

#### Statistical methodology: indicate % of expected drop-outs: the SPRS and 6MWT are normally administered to patients with HSP. It is hypothesized that requiring the additional assessment with the SNAP questionnaire will not cause non-adherence to the study.

**Risk-benefit assessment.**

Possible advantages: Define an instrument that is valid and reliable for use in future rehabilitation clinical trials.

Possible disadvantages and risks: None

Diagnostic-therapeutic alternatives: NP

Study procedures: NP

Precautionary measures to safeguard subjects/patients: Absent

Overall assessment of the risk-benefit ratio: Positive. Zero risk versus benefit of having a useful tool for use in future rehabilitation clinical trials.

REFERENCE

(1)Fink, J.K.(2003). The hereditary spastic paraplegias. Arch Neurol,60,1045-1049.

(2)Appleton, R..(1991). “Pure” and “complicated“ forms of hereditary spastic paraplegia presenting in childhood. Dev Med Child Neurol,33,304-312

(3)Marsden, J.(2012). Muscle paresis and passive stiffness: Key determinants in limiting function in Hereditary and Sporadic Spastic Paraparesis. Gait &Posture,35,266-271

(4)de Niet, M.(2015).Functional effects of botulinum toxin type-A treatment and subsequent stretching of spastic calf muscles: a study in patients with hereditary spastic paraplegia. J Rehabil Med,47,147-153

(5)Bertolucci, F.(2015). Robotic gait training improves skills and quality of life in hereditary spastic paraplegia. NeuroRehabilitation,36,93-99

(6)Zhang, Y.(2014) The effect of hydrotherapy treatment on gait characteristics of hereditary spastic paraparesis patients. Gait & Posture,39,1074-1079

(7)Michels, E(1982). Evaluation and research in physical therapy. Phys Ther,62,828-834

(8)Schule, R(2006). The Spastic Paraplegia Rating Scale (SPRS). Neurology,67,430-434

(9)ATS(2002). Statement: Guidelines for the Six-Minute Walk Test (6MWT). Am J Respir Crit Care Med,166,111-11
