# Supplementary material for: Validation of the Italian version of a patient-reported outcome measure for Hereditary Spastic Paraplegia
Source: PLoS One. 2024 Apr 1;19(4):e0301452. doi: 10.1371/journal.pone.0301452 (PMC10984402; doi:10.1371/journal.pone.0301452)
Supplement: S1 File — (DOCX) [file pone.0301452.s005.docx]

***Validation of the Italian Version of a Patient-Reported Outcome Measure for Hereditary Spastic Paraplegia***

Eleonora Diella^1*^, Maria Grazia D’Angelo^1^, Cristina Stefan^2^, Giulia Girardi^2^,

Roberta Morganti^1^, Andrea Martinuzzi^3^ and Emilia Biffi^1^

***Supplementary material S3***

*We report here the results related to the analysis of HSP-SNAP reliability considering the adult group.*

*The ICC of HSP-SNAP including only adults (N=35) was equal to 0.97, demonstrating high reliability.*

*The mean value of HSP-SNAP for the adult HSP group (N=35) and the adult healthy group (N=35) were equal to 20.3 ±7.2 and 42.5± 6.7, respectively and they significantly differed as in the entire group (U=28.5, p<.001). The mean of adult patients with HSP is a couple of points smaller than the results obtained on the whole group. This result is expected since we previously demonstrated that the age negatively correlates with HSP-SNAP score.*
