# Supplementary material for: Validation of the Italian version of a patient-reported outcome measure for Hereditary Spastic Paraplegia
Source: PLoS One. 2024 Apr 1;19(4):e0301452. doi: 10.1371/journal.pone.0301452 (PMC10984402; doi:10.1371/journal.pone.0301452)
Supplement: Attachment — Submitted filename: Point-byPoint rebuttal_Diella et al_REV2.docx [file pone.0301452.s006.docx]

Dear Editor,

We thank you and the reviewers for your valuable feedback.

We carefully read all the reviewers’ comments and addressed them below.

**Editor Comments:**

The manuscript has been greatly improved; however, some concerns are still pending and need actions.

*We thank the Editor for this comment. We have considered the additional concerns in this revision.*

Reviewers' comments:

Reviewer #1: The authors have addressed most of my suggestions and concerns. It is imperative, though, to clearly state this study's limitations in the abstract, i.e. that the scale is only validated in Italian at this point and that it does not specifically inquire about non-motor symptoms.

*We thank the reviewer for this comment. We have corrected the typo and we have added the limitations of the study in the abstract and as follows:*

*“Conclusion: Although HSP-SNAP does not investigate non-motor symptoms and we validated only its Italian version, it showed good validity and reliability and it could be used in combination with other objective outcome measures for clinical purposes or as endpoints for future clinical rehabilitation studies.”*

Reviewer #2: Dear authors, the paper significantly improved throughout this review process.
Thank you for the hard work!

*Thanks a lot for your comment and work.*

There are only three points left:

1) I disagree with the quotation of reference 18 as stated by reviewer one (I was reviewer #2) so therefore we both disagree and this should still be changed.

*According to your suggestion, we removed reference 18.*

2) The information about the testing adults/children should be added to the supplement. I also follow the suggestions of reviewer #1.

*We thank the reviewer for this comment. We added in the Methods and Results section (Supplementary material S3) the analysis on the group of adults.*

3) With the discussion of the authors in this paragraph, I am delighted.

[...] But I am not totally convinced that endurance is reduced after a resting phase an exercise is conceived with increased spasticity in the beginning phase but then with reduced spasticity and ongoing exercise I am unsure if this really leads to limited endurance or just because gait speed is reduced to a lower distance covered over time. From questionnaires (compare the non-motor study data from Rattay et. al.) the reported maximum gait distance at self-chosen speed is not dramatically reduced compared to healthy controls. [...]

I think this is very interesting point that should be added to the manuscript because this is an ongoing discussion in the field of HSP.

*We thank the reviewer for this fruitful discussion. We have added the following considerations to the Discussion section underling the openness of the point:*

*“However, we are aware of the ongoing debate in the field whether HSP’s gait is actually characterized by reduced endurance or just by reduced walking speed. This is a crucial open point that need further in-depth analysis. Besides, we can’t ignore that fatigue and pain, as reported by Rattay et al [38], are frequent symptoms that need to be screened and treated because they affect health-related quality of life”.*

*Thank you in advance for your attention.*

*Eleonora Diella and Emilia Biffi on behalf of all the authors*
